# Inter-Observer and Intra-Observer Variability in Gross Tumor Volume Delineation of Primary Esophageal Carcinomas Based on Different Combinations of Diagnostic Multimodal Images

**DOI:** 10.3389/fonc.2022.817413

**Published:** 2022-04-01

**Authors:** Fengxiang Li, Yankang Li, Xue Wang, Yingjie Zhang, Xijun Liu, Shanshan Liu, Wei Wang, Jinzhi Wang, Yanluan Guo, Min Xu, Jianbin Li

**Affiliations:** ^1^ Department of Radiation Oncology, Shandong Cancer Hospital and Institute, Shandong First Medical University and Shandong Academy of Medical Sciences, Jinan, China; ^2^ Department of Radiation Oncology, Linyi Cancer Hospital, Linyi, China

**Keywords:** esophageal carcinoma, diagnostic multimodal images, target delineation, intra-observer variability, inter-observer variability

## Abstract

**Background and Purpose:**

This study aimed to investigate inter-/intra-observer delineation variability in GTVs of primary esophageal carcinomas (ECs) based on planning CT with reference to different combinations of diagnostic multimodal images from endoscopy/EUS, esophagography and FDG-PET/CT.

**Materials and Methods:**

Fifty patients with pathologically proven thoracic EC who underwent diagnostic multimodal images before concurrent chemoradiotherapy were enrolled. Five radiation oncologist independently delineated the GTVs based on planning CT only (GTV_C_), CT combined with endoscopy/EUS (GTV_CE_), CT combined with endoscopy/EUS and esophagography (X-ray) (GTV_CEX_), and CT combined with endoscopy/EUS, esophagography, and FDG-PET/CT (GTV_CEXP_). The intra-/inter-observer variability in the volume, longitudinal length, generalized CI (CI_gen_), and position of the GTVs were assessed.

**Results:**

The intra-/inter-observer variability in the volume and longitudinal length of the GTVs showed no significant differences (*p>*0.05). The mean intra-observer CI_gen_ values for all observers was 0.73 ± 0.15. The mean inter-observer CI_gen_ values for the four multimodal image combinations was 0.67 ± 0.11. The inter-observer CI_gen_ for the four combined images was the largest, showing significant differences with those for the other three combinations. The intra-observer CI_gen_ among different observers and inter-observer CI_gen_ among different combinations of multimodal images showed significant differences (*p*<0.001). The intra-observer CI_gen_ for the senior radiotherapists was larger than that for the junior radiotherapists (*p*<0.001).

**Conclusion:**

For radiation oncologists with advanced medical imaging training and clinical experience, using diagnostic multimodal images from endoscopy/EUS, esophagography, and FDG-PET/CT could reduce the intra-/inter-observer variability and increase the accuracy of target delineation in primary esophageal carcinomas.

## Highlights

- There is large variability in target volume delineation for esophageal carcinoma.
- Evaluation of inter-/intra-observer delineation variability based on diagnostic multimodal imaging.
- Multimodal diagnostic image combinations can reduce the intra-/inter-observer variability and increase delineation accuracy.

## Introduction

Esophageal carcinoma is the seventh most commonly diagnosed cancer and the third leading cause of cancer deaths worldwide in 2018 (1). Preoperative and definitive chemoradiation therapies have played a key role in the treatment of esophageal carcinoma (2–5). The proportion of residual esophageal carcinoma after chemoradiation is significantly correlated with locoregional and distant failure (6–9). Reliable delineation of the target gross tumor volume (GTV) is required for accurate radiation dose delivery and successful radiation therapy (10, 11). There is generally large variability in the target volume delineation for esophageal carcinoma, which might be primarily derived from the geometric uncertainties of different images and inherent variability among different observers based on the studies on other malignancies (12, 13).

Conventional three-dimensional CT (3DCT) has been the workhorse modality used to delineate the esophageal tumor target volume. However, it is difficult to determine the proximal and distal extension of tumors and differentiate the layers of the esophageal wall (14–16). An esophagography has shown a higher accuracy in assessing the tumor length (59% of cases, compared with 32% with CT), with tumor morphology influencing the accuracy (14–16). Although endoscopy and endoscopic ultrasonography (EUS) might present the tumor length more accurately (17, 18), it is difficult to transform the imaging to radiotherapy (RT) planning (19). Fluorine-18 fluorodeoxyglucose positron emission tomography/computed tomography (18F-FDG-PET/CT) has proved useful for diagnosing and staging esophageal carcinoma. However, there is limited evidence supporting the validity of FDG-PET/CT for target volume delineation (20, 21). The false-positive FDG uptake in areas of inflammation reduces the specificity of tumor extent visualization (22). Therefore, the combination of multimodal images is critical for determining the GTV of esophageal cancer (EC) accurately. Several studies have focused on the inter-observer variability of target volume delineation in FDG-PET/CT compared with pure CT imaging (10, 23). As CT imaging has proved indispensable for the visualization/detection of esophageal tumors, the use of multimodality imaging including esophagography, endoscopy/EUS and FDG-PET/CT for target volume delineation has not received sufficient attention.

In general, patients scheduled to receive radiotherapy or chemoradiotherapy undergo diagnostic multimodal imaging including enhanced CT, endoscopy/EUS, esophagography, or FDG-PET/CT. In clinical practice, radiation oncologists generally delineate the target volumes based on the planning CT images, with reference to various preexisting diagnostic images. However, the outcome of using different combinations of diagnostic multimodal images on the inter-observer and intra-observer delineation variability remains unclear. The purpose of this study was to investigate the inter-observer and intra-observer delineation variability in the GTVs of primary esophageal tumors with reference to different combinations of multimodal images from endoscopy/EUS, esophagography, and FDG-PET/CT. This study indicated the influence of the addition of different multimodal images on the GTVs delineation variability, which may contribute to making clinical decision on acquire different multimodal images.

## Materials and Methods

### Patient Selection and Characteristics

This study was approved by the institutional research ethics board and informed consent has been obtained from the participants involved. Fifty-one patients with pathologically proven thoracic EC who had undergone preoperative or definitive concurrent chemoradiotherapy between May 2015 and June 2017 at the institutional hospital were enrolled. Among the selected patients, there were seventeen cases each of upper, middle, and lower EC. One patient with lower EC was excluded due to the lack of PET-CT imaging data. All patients underwent a diagnostic imaging examination that included an endoscopy/EUS, esophagography, and FDG-PET/CT before receiving chemoradiotherapy. The average time for acquiring the diagnostic images was within the two-week period before chemoradiotherapy. **Table 1** presents the patient characteristics.

**Table 1:** Patient’s characteristics.

Characteristics	Number
Sex, n (%)	
M	40 (80%)
F	10 (20%)
Age, median, y (range)	63 (44-88)
Tumor histology, n (%)	
Squamous cell carcinoma	50 (100%)
SUV_max_, mean, median, y (range)	17.1, 15.2 (2.8~49.5)
TNM* stage, n (%)	
T_2_N_0-2_M_0_	4 (8%)
T_3_N_1-3_M_0-1_	34 (68%)
T_4a_N_0-2_M_0-1_	12 (24%)
Tumor location, n (%)	
Upper	17 (34%)
Mid-	17 (34%)
Distal	16 (32%)

### Multimodal Imaging

Endoscopy/EUS examination: All patients underwent diagnostic endoscopy examinations using an electronic gastroscope (Olympus GIF-Q260J) before treatment. Seven patients did not undergo EUS examinations due to esophageal stenosis. The ultrasonic probe (Olympus EVIS EUS EU-ME2) was inserted into the patient’s esophagus along the track of the biopsy forceps to detect the depth of tumor infiltration in the esophageal wall and the extent of proximal and distal tumor infiltration. The distances from the proximal and distal ends of the tumor to the incisors were recorded.

Esophagography (X-ray) image acquisition: Esophagography was performed before treatment using a digital radiography machine (Siemens Luminos dRT Max). All barium examinations were performed under fasting conditions, followed by a standard protocol (drinking 200 ml of diluted barium, in the upright, supine, and prone positions, with and without the gas powder).

PET/CT image acquisition: The PET-CT scan was performed within the two-week period prior to the planning CT scan as a part of the routine diagnostic management for EC. An 18F-FDG PET/CT scan of the chest was performed with an integrated PET/CT system (Philips Gemini TF Big Bore). The PET images were reconstructed with the CT-derived attenuation correction using an ordered subset expectation maximization algorithm with post-reconstruction Gaussian filtering, with a full width at half maximum of 5 mm.

Planning CT image acquisition: During the simulation, all patients were immobilized using a thermoplastic mask in the supine position with the arms placed along the side of the body. Each patient underwent an enhanced planning CT scan of the thoracic region on a 16-slice CT scanner (Philips Brilliance Bores CT) under free-breathing conditions. The planning CT images were reconstructed using a thickness of 3 mm and subsequently transferred to an Eclipse treatment planning system (Varian Eclipse 11).

### Target Volume Delineation

A treatment planning system (Eclipse; Varian Medical Systems, Inc., Palo Alto, CA, USA) was used to contour the GTVs of the primary EC. The visualization parameter for delineation included the mediastinal window set to +40/400 HU. Before contouring, some clinical information such as the physical examination, pathological findings, and diagnostic CT image data were made available to the observers, while they were blind to the diagnostic endoscopy/EUS, esophagography, and FDG-PET/CT data. If the positive lymph nodes could not be separated from the primary tumor visually, they were delineated together with the primary tumor.

**Figure 1:**
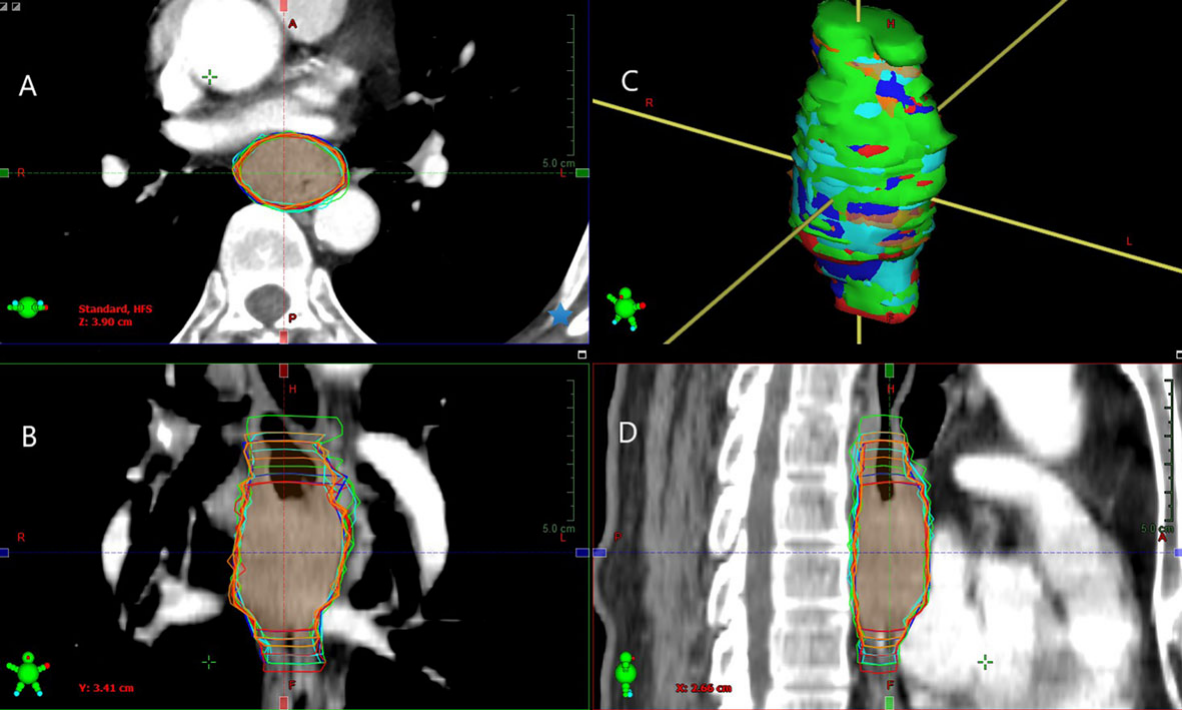
Example of GTVs delineated based on different combinations of multimodal images by observer 1 (green segment), observer 2 (red segment), observer 3 (blue segment), observer 4 (orange segment), and observer 5 (cyan segment) in tansversal **(A)**, frontal **(B)**, surface **(C)**, and sagittal **(D)** planes for one patient (Patient 5). Inter-/intra-observer variability in the volume and longitudinal length on different combinations of multimodal images exhibiting significant differences.

Five radiation oncologists (observers), who were blind to the diagnostic endoscopy/EUS, esophagography, and FDG-PET/CT patient data, were asked to independently delineate the GTVs with reference to different combinations of the multimodal images, including planning CT only (GTV_C_), CT combined with endoscopy/EUS (GTV_CE_), CT combined with endoscopy/EUS and esophagogram (X-ray) (GTV_CEX_), and CT combined with endoscopy/EUS, esophagogram, and FDG-PET/CT (GTV_CEXP_) (**Figure 1**). All observers were blind to the contours delineated by the other oncologists and their own former/previous contours. Observers 1 and 2 with clinical experience within five years were regarded as junior observers, while observers 3, 4, and 5 with more than ten years of clinical experience were regarded as senior observers. All contours were delineated in about two years. A delay of at least two months existed between each contouring of the tumor to eliminating a recall of the previous contouring for observers 1, 2, 3, and 5. The time interval for observer 4 was only one month, as the former observer 4 dropped out of the delineation process due to parturition.

### Inter-/Intra-Observer Variability Analysis

Inter-/intra-observer variability in the volume, longitudinal length, generalized conformity index (CI_gen_), and position of the GTVs was assessed. The intra-observer variability can be generally regarded as the variability of the same observer when re-contouring a single case. However, in this study, it is defined as the variability of the contours on the four multimodal imaging/image combinations for one observer (23).

The mean volume and longitudinal length of the GTVs based on different multimodal imaging combinations for different observers were calculated. The inter-observer variability in the volume and longitudinal length on different multimodal imaging, combinations and the intra-observer variability for different observers were measured. The tumor length was measured using CT, endoscopy/EUS (43 cases), esophagography, and FDG-PET/CT, with the difference between the tumor length and corresponding longitudinal length of the GTVs subsequently evaluated.

Conformity index (CI) was defined as the ratio of the common volume to encompassing volume (13, 24). The generalized CI (CI_gen_) was used to assess the overall consistency of all volume combinations delineated by different observers on the same imaging-modality combination, and that delineated by the same observer on different imaging-modality combinations. The formula is given by (13, 25):


$$CI_{gen}=\frac{\sum_{pairsij}\left|A_{i}\cap A_{j}\right|}{\sum_{pairsij}A_{i}\cup A_{j}}$$

CI_gen_ is a good parameter for revealing the difference in the volumes delineated based on the size, shape, and location (10, 23). The use of CI_gen_ tends to decrease the bias in the number of delineations (13). The lower is the CI_gen_ value for the same imaging-modality combination, the greater is the inter-observer variability. Similarly, a lower CI_gen_ for the same observer suggests a greater intra-observer variability.

In addition, the x (right-left), y (anterior-posterior), and z (superior-inferior) axes of the center of mass (COM) of the volume were measured. The centroid shifts between the different volumes were then obtained. Finally, the three dimensional (3D) centroid shifts were calculated using the followed equation (24, 26):


$$3D\;centroid\;shifts=\sqrt{\Delta x^{2}+\Delta y^{2}+\Delta z^{2}}$$

### Statistical Analysis

Statistical analysis was performed using the SPSS software package (SPSS 25.0). All the data had an approximately normal distribution. The one-way ANOVA test was applied to detect the inter-/intra-observer variability in the volume, longitudinal length, CI_gen_, and position of the GTVs among different observers and different multimodal imaging combinations. The paired *t*-test was used to compare the volume, longitudinal length, CI_gen_, and position of the GTVs between two observers or two multimodal imaging combinations. A P<0.05 was considered significant.

## Results

### *GTV* Volume

**Table 2** shows the primary GTV delineated based on four different multimodal imaging combinations for each observer. No significant inter-observer differences in the volume were observed for GTV_C_, GTV_CE_, GTV_CEX_, or GTV_CEXP_ (*p*= 0.904, 0.987, 0.984, and 0.97, respectively). The intra-observer variability in the volume of the GTVs derived from four different multimodal imaging combinations for observers 1–5 also showed no significant differences (*p*= 0.926, 0.997, 0.908, 0.943, and 0.99, respectively). However, the paired comparisons indicated significant differences in the GTV volume between observers 1 and 2, observers 1 and 4, and observers 3 and 4 (*t*= 3.154, 6.368, and 3.342, *p*= 0.002, <0.001, and 0.001, respectively). Approximate statistical differences in the GTV volume were found between observers 1 and 3, and observers 2 and 4 (*t*= 3.342 and 1.869, *p*= 0.061 and 0.063, respectively).

**Table 2:** The volume and longitudinal length of GTVs based on different combinations of multimodal imaging for different observers.

Parameter		Observer 1	Observer 2	Observer 3	Observer 4	Observer 5	Mean±SD
**GTV_C_**	Volume(cm^3^)	37.57 ± 26.68	33.78 ± 27.42	36.42 ± 26.91	33.48 ± 28.07	33.28 ± 26.87	34.91 ± 27.19
	Length(mm)	5.7 ± 2.6	5.3 ± 2.5	6.3 ± 2.5	5.7 ± 2.6	5.3 ± 2.3	5.7 ± 2.5
**GTV_CE_**	Volume(cm^3^)	34.06 ± 25.90	34.47 ± 26.65	36.33 ± 27.10	32.68 ± 25.79	35.13 ± 27.50	34.68 ± 26.82
	Length(mm)	5.7± 2.5	5.4 ± 2.2	6.3 ± 2.4	5.8 ± 2.4	5.7 ± 2.3	5.8 ± 2.4
**GTV_CEX_**	Volume(cm^3^)	35.43 ± 25.79	35.00 ± 26.40	33.57 ± 26.68	34.50 ± 26.84	35.03 ± 28.37	34.34 ± 26.81
	Length(mm)	5.6 ± 2.2	5.6 ± 2.2	5.5 ± 2.3	5.6 ± 2.2	5.6 ± 2.5	5.6 ± 2.3
**GTV_CEXP_**	Volume(cm^3^)	36.30 ± 27.41	34.17 ± 26.19	33.48 ± 27.13	33.28 ± 26.67	36.73 ± 28.27	35.04 ± 27.17
	Length(mm)	6.1± 2.5	5.7 ± 2.2	5.5 ± 2.3	5.8 ± 2.4	5.8 ± 2.3	5.8 ± 2.3
**Mean ± SD**	Volume(cm^3^)	35.84 ± 26.45	34.36 ± 26.67	34.95 ± 27.20	33.52 ± 26.91	35.04 ± 27.75	34.37 ± 27.29
	Length(mm)	5.8 ± 2.4	5.5 ± 2.3	5.9 ± 2.4	5.7 ± 2.4	5.6 ± 2.4	5.7 ± 2.4

Gross target volumes (GTV) delineated on planning CT only (GTV_C_), CT combined with endoscopy/EUS (GTV_CE_), CT combined with endoscopy/EUS and esophagography (X-ray) (GTV_CEX_), and CT combined with endoscopy/EUS, esophagography, and FDG-PET/CT (GTV_CEXP_).

### Esophageal Tumor Length

**Table 3** shows the mean tumor lengths measured by CT, endoscopy/EUS, esophagography, and FDG-PET/CT. No significant differences were found between any two image-based tumor lengths. **Table 3** presents the mean longitudinal lengths measured by the five observers corresponding to GTV_C_, GTV_CE_, GTV_CEX_, and GTV_CEXP_. The mean longitudinal length for GTV_CEXP_ was larger than the tumor length measured by FDG-PET/CT (*p*=0.0035). The intra-observer variability in the longitudinal length of the GTVs based on four multimodal imaging combinations for observers 1–5 showed no significant differences (*p*= 0.751, 0.794, 0.115, 0.962, and 0.753, respectively). **Table 2** shows the tumor lengths measured based on the four different multimodal imaging combinations for each observer. No significant inter-observer differences in the longitudinal length were recorded for GTV_C_, GTV_CE_, GTV_CEX_, and GTV_CEXP_ (*p*= 0.286, 0.503, 0.997, and 0.749, respectively). The two-related-samples tests indicated significant differences in the longitudinal lengths of the four GTVs between observers 1 and 2 (*t*=2.776, *p*=0.006), observers 1 and 5 (*t*=1.98, *p*=0.049), observers 3 and 2 (*t*=−3.166, *p*=0.002), and observers 3 and 5 (*t*=2.992, *p*=0.003).

**Table 3:** Comparison the tumor length measured by CT, endoscopy/EUS, esophagography, and FDG-PET/CT with the mean longitudinal length measured by five observers for GTV_C_, GTV_CE,_ GTV_CEX,_ and GTV_CEXP_.

Imaging modality		CT	Endoscopy/EUS	Esophagography	PET-CT
** Tumor length(cm)**		5.5 ± 2.2	5.1 ± 2.0	5.3 ± 2.0	5.4 ± 2.2
**Target volume**		**GTV_C_**	**GTV_CE_**	**GTV_CEX_**	**GTV_CEXP_**
** Longitudinal length(cm)**		5.7 ± 2.5	5.8 ± 2.4	5.6 ± 2.3	5.8 ± 2.3
**Paired comparison**	**t-value**	-0.704	-1.759	-1.272	-2.172
	**p-value**	0.485	0.086	0.209	0.035

Gross target volumes (GTV) delineated on planning CT only (GTV_C_), CT combined with endoscopy/EUS (GTV_CE_), CT combined with endoscopy/EUS and esophagography (X-ray) (GTV_CEX_), and CT combined with endoscopy/EUS, esophagography, and FDG-PET/CT (GTV_CEXP_).

### Generalized CI (CI_gen_)

**Table 4** lists the mean CI_gen_ values for the four GTVs derived from different multimodal imaging combinations (mean intra-observer CI_gen_) for each observer. The mean intra-observer CI_gen_ values for all observers was 0.73 ± 0.15. The mean intra-observer CI_gen_ was the largest for observer 4, exhibiting significant differences with that for the other observers. The mean intra-observer CI_gen_ for observer 1 was the lowest, exhibiting significant differences with that for observers 3, 4, and 5. The mean intra-observer CI_gen_ among different observers was statistically significant (*F*=32.493, *p*<0.001). **Table 5** lists the mean CI_gen_ values for the five GTVs derived from different observers (mean inter-observer CI_gen_) for each multimodal imaging combination. The mean inter-observer CI_gen_ values for the four multimodal imaging combinations was 0.67 ± 0.11. The mean inter-observer CI_gen_ was the largest for the fourth multimodal imaging combination, which exhibited significant differences with that for the other three combinations. The mean inter-observer CI_gen_ among the different multimodal imaging combinations showed a significant difference (*F*=6.872, *p*<0.001).

**Table 4:** The CI_gen_ values and 3D centroid shifts (Mean ± SD) of the four GTVs derived from different combinations of multimodal imaging for each observer.

Parameter	Observer 1	Observer 2	Observer 3	Observer 4	Observer 5
**CI_gen_**	0.68 ± 0.12	0.69 ± 0.14	0.75 ± 0.15	0.80 ± 0.15	0.74 ± 0.17
**Paired t-test** **(p value)**	0.173 (vs Obs2)	–	–	–	–
	<0.001 (vs Obs3)	<0.001 (vs Obs3)	–	–	–
	<0.001 (vs Obs4)	<0.001 (vs Obs4)	<0.001 (vs Obs4)	–	–
	<0.001 (vs Obs5)	<0.001 (vs Obs5)	0.264 (vs Obs5)	<0.001 (vs Obs5)	–
**CI (G_C_, G_CEXP_)**	0.66 ± 0.12	0.70 ± 0.18	0.75 ± 0.13	0.78 ± 0.17	0.73 ± 0.18
**CI (G_C_, G_CE_)**	0.69 ± 0.15	0.68 ± 0.14	0.77 ± 0.15	0.84 ± 0.15	0.78 ± 0.17
**Paired t-test t**	-1.718	1.247	-0.848	-2.592	-2.666
**(p value)**	0.092	0.218	0.4	0.013	0.01
**3D shifts(mm)**	3.69 ± 4.47	4.34 ± 4.24	3.84 ±3.93	2.85 ± 4.24	3.65 ± 6.19
**Paired t-test** **(p value)**	0.023 (vs Obs2)	–	–	–	–
	0.572 (vs Obs3)	0.077 (vs Obs3)	–	–	–
	0.005 (vs Obs4)	<0.001 (vs Obs4)	0.001 (vs Obs4)	–	–
	0.911 (vs Obs5)	0.084 (vs Obs5)	0.59 (vs Obs5)	0.039 (vs Obs5)	–

Gross target volumes (GTV) delineated on planning CT only (G_C_), CT combined with endoscopy/EUS (G_CE_), and CT combined with endoscopy/EUS, esophagography, and FDG-PET/CT (G_CEXP_). The CI between G_C_ and G_CEXP_ [CI (G_C_, G_CEXP_)], the CI between G_C_ and G_CE_ [CI (G_C_, G_CE_)].

**Table 5:** The CI_gen_ values and 3D centroid shifts (Mean ± SD) of the five GTVs delineated by different observers based on each combinations of multimodal imaging.

Parameter	GTV_C_	GTV_CE_	GTV_CEX_	GTV_CEXP_
**CI_gen_**	0.66 ± 0.13	0.66 ± 0.12	0.67 ± 11	0.69 ± 0.10
**Paired t-test** **(p value)**	0.443 (vs G_CE_)	–	–	–
	<0.088 (vs G_CEX_)	0.269 (vs G_CEX_)	–	–
	<0.001 (vs G_CEXP_)	<0.001 (vs G_CEXP_)	<0.001 (vs G_CEXP_)	–
**3D shifts (mm)**	3.78 ± 4.04	3.78 ± 3.79	3.98 ± 5.03	3.68 ± 5.94
**Paired t-test** **(p value)**	0.981 (vs G_CE_)	–	–	–
	0.463 (vs G_CEX_)	0.463 (vs G_CEX_)	–	–
	0.762 (vs G_CEXP_)	0.744 (vs G_CEXP_)	0.218 (vs G_CEXP_)	–

Gross target volumes (GTV) delineated on planning CT only (GTV_C_), CT combined with endoscopy/EUS (GTV_CE_), CT combined with endoscopy/EUS and esophagography (X-ray) (GTV_CEX_), and CT combined with endoscopy/EUS, esophagography, and FDG-PET/CT (GTV_CEXP_).

### Three-Dimensional (3D) Centroid Shifts

**Table 4** lists the mean 3D centroid shifts of the four GTVs derived from different multimodal imaging combinations (mean intra-observer 3D centroid shifts) for each observer. The mean intra-observer 3D centroid shifts for all observers was 3.67 ± 4.62 mm. The mean intra-observer 3D centroid shifts for observer 4 showed significant differences compared with the other observers. The mean intra-observer 3D centroid shifts among different observers was significant (*F*=3.898, *p*=0.004). **Table 5** presents the 3D centroid shifts of the five GTVs derived from different observers (mean inter-observer 3D centroid shifts) for each multimodal imaging combination. The mean inter-observer 3D centroid shifts for all four multimodal imaging combinations was 3.81 ± 4.7 mm. The mean inter-observer 3D centroid shifts among the different multimodal imaging combinations showed no significant difference (*F*=0.327, *p*=0.806).

## Discussion

Uncertainties in volume delineation for esophageal carcinomas is a well-recognized potential cause of treatment failure in radiotherapy (27, 28). Minimizing the inter-/intra-observer delineation variability in volume delineation is regarded as an effective alternative method to define the GTV accurately (29, 30), since the gold standard of a pathological reference volume is rarely attainable (31, 32). The significance of quantifying the degree of variability or uncertainty in volume delineation is that the resulting impact on dosimetry and clinical outcomes (29, 30).

Accurate target delineation for esophageal cancer is often restricted by the poor discriminative value of current imaging modalities (23), particularly CT, and the inability to relate diagnostic endoscopy/EUS, esophagography, or FDG-PET/CT information to the panning CT images (13–17, 23). However, reasonable pretreatment staging assessments are essential to determine a rational treatment strategy. In each patient with newly diagnosed esophageal cancer, the acquired diagnostic imaging information should identify the feasibility of delineating the GTVs of the primary based on the planning CT image with reference to the above-mentioned information. In this study, the geometric features of the GTVs derived from different observers and different planning CT image combinations were compared with the diagnostic imaging information. Furthermore, the value of the different planning CT image combinations in conjunction with diagnostic imaging information was evaluated for tumor delineation in esophageal carcinoma.

The results of this study showed no statistically significant inter-observer differences in the esophageal volume estimation based on different combinations of the CT, endoscopy/EUS, esophagography, and FDG-PET/CT data (**Table 2**). For a particular multimodal imaging combination, different observers reported similar estimates for the GTV based on a similar knowledge of multimodal imaging. Moreover, for each observer, the volumes of the four GTVs delineated on different multimodal imaging combinations showed no significant differences. This indicates that the GTV volume assessments on different multimodal imaging combinations did not transform/change for the same observer. The data presented here is similar to the results reported in other literature (33, 34). However, Choi et al. (13) reported that the number of observers and number of observations made might affect the level of significance. In this study, many significant differences were observed in the GTV volume between different observers in the pairwise comparisons. Therefore, inter-observer variation in the target delineation could not be revealed/identified by merely comparing the volumes of the GTVs.

Similar to the observed variability in the volumes of the GTVs, the inter-observer and intra-observer variability in the longitudinal length showed no statistically significant differences (**Table 3**). However, some significant differences between different observers were identified in the pairwise comparisons. The main reason behind these differences might be a different understanding of the procedure of determining the tumor length on multimodal imaging by different observers. Radiation oncologists have always found the procedure to determine the proximal and distal extension of esophageal carcinoma based on different images challenging. Conventional images from CT, endoscopy/EUS, and esophagography, and MRI or FDG-PET/CT have their share of advantages and limitations for determining the tumor length (14–17, 22, 35, 36). It is critical to familiarize radiation oncologists with these advantages and limitations before selecting the different image combinations. In this study, the tumor length determined by the multimodal images tended to be larger than that measured by a single image. In particular, the longitudinal length of GTV_CEXP_ was significantly larger than the tumor length measured by FDG-PET/CT. Therefore, the use of the multimodal images to determine the target length contributes to reducing the limitation of a single image, and improving the accuracy of target delineation; however, this is based on the precondition that the observers develop a good knowledge of the features of the multimodal images *via* unified training.

The CI_gen_ values for GTV_C_, GTV_CE_, GTV_CEX_, and GTV_CEXP_ for each observer represent the intra-observer variations, which include the random and inherent variations derived from different multimodal imaging combinations for the same observer. Here, the mean CI_gen_ for intra-observer variability (0.73) was larger than that for inter-observer variability (0.67). This indicates that the intra-observer variability in delineating esophageal tumors was lower than the inter-observer variability, which shows agreement with the results reported in other studies (33, 34). Machiels et al. (33) reported the mean CI_gen_ values for intra-observer delineation variability and inter-observer variability in ten patients without endoscopically implanted fiducial markers versus those with markers to be 0.54 versus 0.68 and 0.68 versus 0.75, respectively. Vollenbrock et al. (34) reported the mean CI_gen_ over six patients as 0.68 on FDG-PET/CT, 0.66 on T_2_w-MRI, and 0.68 on T_2_w+DW(diffusion-weighted)-MRI. Compared with the above studies, fifty patients with upper, middle, and lower thoracic esophageal carcinoma were enrolled in this study. Moreover, different multimodal imaging combinations, including CT, endoscopy/EUS, esophagography, and FDG-PET/CT, were employed to eliminate any bias from a single imaging technique.

In addition, the CT is a basic image (GTV_C_). CT combined with endoscopy/EUS is a simple combination(GTV_CE_), while CT combined with endoscopy/EUS, esophagography, and FDG-PET/CT (GTV_CEXP_) is regarded as an effective alternative method to define the GTV accurately (**Table 5**). Therefore, The CI between GTV_C_ and GTV_CEXP_, was significantly less than the CI between GTV_C_ and GTV_CE_ for all observers (*t*= -3.018, *p* = 0.003), which suggested that a comprehensive combination of multimodal images was more conducive to influence the target delineation compared a simple combination.

In the ANOVA analysis, the intra-observer CI_gen_ for the GTVs derived from different multimodal imaging combinations among the five observers was statistically significant (*p<*0.001). The intra-observer CI_gen_ for the senior radiation oncologists (observers 3, 4, and 5) was larger than that for the junior radiation oncologists (observers 1 and 2). An optimum intra-observer CI_gen_ was obtained for the senior radiotherapist who spent minimal time delineating the GTVs (observer 4) (**Table 4**). The senior radiotherapists, who were generally familiar with the multimodal imaging features for distinguishing the tumors from the normal structures and the location subject to relapse, might not be easily affected when only a single imaging modality is used/available for target contouring (37, 38). In addition, the shorter repeating delineation intervals did not eliminate the record of previous delineations, which might have improved the consistency of the target delineation. This suggests that background knowledge in medical imaging, clinical experience, and repeating delineation intervals might affect the intra-observer variability of the target CI_gen_. Strengthening the target delineation and medical imaging knowledge training contributes to improve the accuracy of target delineation for EC.

**Figure 2:**
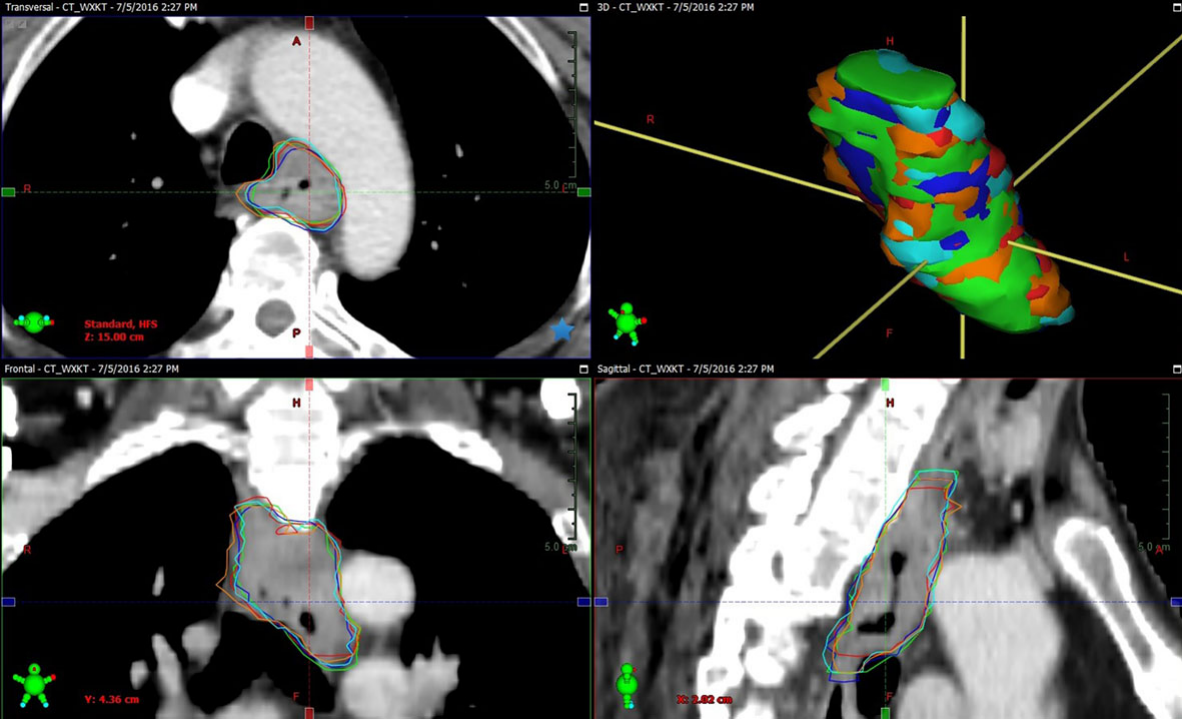
Example of GTVs delineated based on CT combined with endoscopy/EUS, esophagogram, and FDG-PET/CT (GTV_CEXP_) by observer 1 (green segment), observer 2 (red segment), observer 3 (blue segment), observer 4 (orange segment), and observer 5 (cyan segment) in tansversal, frontal, surface, and sagittal planes for one patient (Patient 10). The volume and longitudinal length of GTV_CEXP_ delineated on CT combined with endoscopy/EUS, esophagogram, and FDG-PET/CT exhibiting a good consistency.

While the inter-observer CI_gen_ calculated for the different multimodal imaging combinations did not increase for the combined CT and endoscopy/EUS data, as compared with CT only, CI_gen_ tended to increase for the combined CT and endoscopy/EUS and esophagography information (*p*=0.088). Furthermore, the addition of FDG-PET/CT to the endoscopy/EUS, and esophagography data significantly improved the inter-observer CI_gen_. The use of multimodal imaging, including CT, endoscopy/EUS, esophagography, and FDG-PET/CT, for target delineation reduced the inter-observer variability. **Figure 2** showed example of GTVs delineated based on CT combined with endoscopy/EUS, esophagogram, and FDG-PET/CT (GTV_CEXP_) by five oncologists, and the volume and longitudinal length of GTV_CEXP_ exhibited a good consistency. The effect of FDG-PET/CT on the intra- and inter-observer variability of target volume delineation in patients with gastro-esophageal cancer remains controversial. Vesprini et al. (39) reported that the combined use of FDG-PET/CT based on CT for GTV delineation significantly decreased both intra- and inter-observer variability, while Schreurs et al. (40) did not find PET/CT to have a significant effect on the inter-observer variability. Therefore, besides FDG-PET/CT, the additional use of endoscopy/EUS and esophagography for target delineation might prove beneficial in reducing the inter-observer variability. Recent studies have shown that the use of endoscopically implanted fiducial markers and MRI might reduce the variability of target volume delineation (33, 34). The use of multimodal imaging has proved increasingly valuable in improving the accuracy of target definition in esophageal carcinoma.

The target conformity index (CI) is mainly influenced by positional and morphological difference of targets. Here, the intra-observer variability in the 3D centroid shifts of the GTVs among different observers showed a significant difference. In the case of no significant variability in the volume of the GTVs, the intra-observer variability in the position could have mainly contributed to the statistical significance in the CI_gen_ value. The intra-observer variability in the shape also tends to affect the intra-observer CI_gen_. The inter-observer variability in the 3D centroid shifts and volume of the GTVs showed no significant differences, suggesting that the inter-observer variability in the shape had a critical influence on the inter-observer CI_gen_. Thus, this study indirectly implies that using different multimodal image combinations might transform/change different observers’ visual perception of tumors. In addition, we found the 3D centroid shifts were 3-4mm either between the observers themselves or between the observers. Therefore, whether it is necessary to expand an extra margin to include this error is a clinical problem and deserves further thinking.

## Conclusion

In conclusion, for radiation oncologists with advanced medical imaging training and clinical experience, the use of diagnostic multimodal images from endoscopy/EUS, esophagography, and FDG-PET/CT for target delineation based on planning CT reduced the intra- and inter-observer variability and increased the accuracy of target delineation in primary thoracic esophageal carcinomas. The use of the combination of multimodal images would reduce uncertainties in volume delineation for esophageal carcinomas, and potentially increase the success rate of radiotherapy. We also found the inter/intra observer variability in the 3D centroid shifts of GTVs were about 3-4mm, whether it is necessary to expand an extra margin to include this error deserves further thinking.

## Data Availability Statement

The original contributions presented in the study are included in the article/supplementary material. Further inquiries can be directed to the corresponding authors.

## Ethics Statement

The studies involving human participants were reviewed and approved by Shandong Cancer Hospital and Institute. The patients/participants provided their written informed consent to participate in this study.

## Author Contributions

FL and YL contributed to the study design, the patient enrollment, the data statistics and analysis, and writing the manuscript. JL participated in the study design. XW contributed to the patient enrollment. YZ, XL, SL, JW, and MX contributed to the delineation. WW and YG made important contributions in collecting the data and revising the content. All authors contributed to the article and approved the submitted version.

## Funding

This work was supported by Shandong Cancer Hospital and Institute, China. National Natural Science Foundation of China (817732870); Beijing Medical Award Foundation (YXJL-2020-0785-0616); Taishan Scholars Program of Shandong Province (NO.ts 20190982); Wu Jieping Medical Foundation (320.6750.2021-02-79).

## Conflict of Interest

The authors declare that the research was conducted in the absence of any commercial or financial relationships that could be construed as a potential conflict of interest.

## Publisher’s Note

All claims expressed in this article are solely those of the authors and do not necessarily represent those of their affiliated organizations, or those of the publisher, the editors and the reviewers. Any product that may be evaluated in this article, or claim that may be made by its manufacturer, is not guaranteed or endorsed by the publisher.
